# Supplementary material for: The causal relationship between the human gut microbiota and pyogenic arthritis: a Mendelian randomization study
Source: Front Cell Infect Microbiol. 2024 Nov 26;14:1452480. doi: 10.3389/fcimb.2024.1452480 (PMC11629706; doi:10.3389/fcimb.2024.1452480)
Supplement: Supplementary file 1 [file DataSheet1.zip › mendelian test/ebi-a-GCST90027632.csv_leave_one_out.pdf]

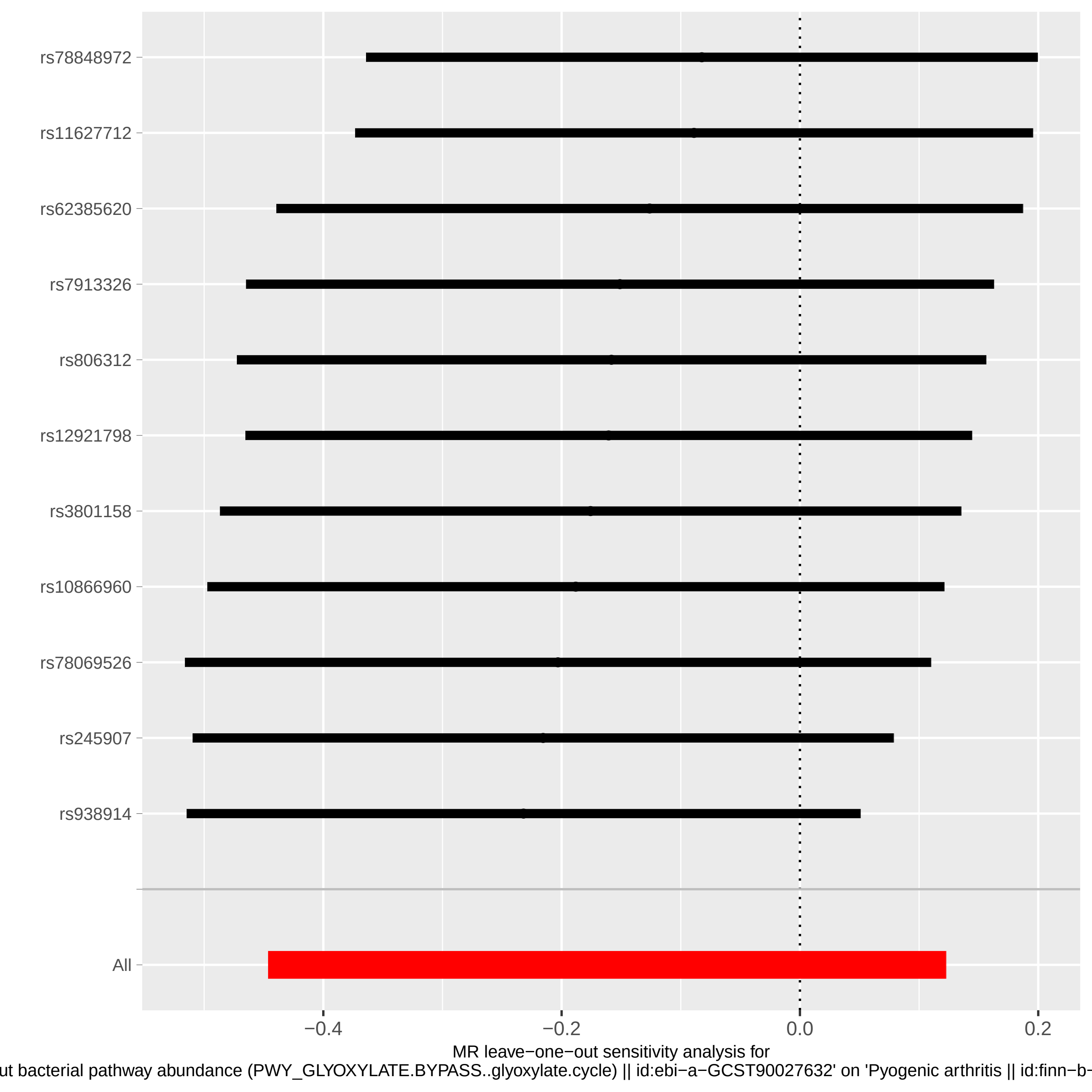
rs78848972
rs11627712
rs62385620
rs7913326
rs806312
rs12921798
rs3801158
rs10866960
rs78069526
rs245907
rs938914
All
−0.4
−0.2
0.0
0.2
MR leave−one−out sensitivity analysis for
ut bacterial pathway abundance (PWY_GLYOXYLATE.BYPASS..glyoxylate.cycle) || id:ebi−a−GCST90027632' on 'Pyogenic arthritis || id:finn−b−
